# Ultrasound combined with Ki-67 to construct the prognostic model for radioactive iodine therapy outcomes in Graves’ disease patients

**DOI:** 10.1530/EC-23-0429

**Published:** 2024-01-16

**Authors:** Yuegui Wang, Liwei Hong, Caiyun Yang, Guorong Lv, Kangjian Wang, Xuepeng Huang, Haolin Shen

**Affiliations:** 1 Department of Ultrasound, Zhangzhou Affiliated Hospital of Fujian Medical University, Zhangzhou, Fujian, China; 2 Department of Nuclear Medicine, Zhangzhou Affiliated Hospital of Fujian Medical University, Zhangzhou, Fujian, China; 3 School of Clinical Medicine, Fujian Medical University, Fuzhou, Fujian, China; 4 Quanzhou Medical College, Quanzhou, Fujian, China

**Keywords:** Ki-67, Graves’ disease, radioactive iodine, ultrasound

## Abstract

The aim of this study was to develop a prognostic model for radioactive iodine (RAI) therapy outcome in patients with Graves‘ disease. We enrolled 127 patients. Information on RAI therapy, ultrasound indexes of thyroid, and other lifestyle factors was collected. The competing risk model was used to estimate the multivariable-adjusted hazard ratios (HRs) and 95% confidence intervals (CIs) for nonhealing or recurrence of hyperthyroidism (NHRH). The performance of the model was assessed by receiver operator characteristic analysis and the Brier score and internally validated by bootstrap resampling. Then, a nomogram was developed. Forty-one cases (32.2%) of NHRH were documented. Positive Ki-67 expression, a higher dose of per-unit thyroid volume, and females showed lower risks of NHRH (all *P* < 0.05). The HR values (95% CI) were 0.42 (0.23, 0.79), 0.01 (0.00, 0.02), and 0.47 (0.25, 0.89), respectively. The bootstrap validation showed that the model had the highest accuracy and good calibration for predicting cumulative risk of NHRH at 180 days after RAI therapy (AUC = 0.772; 95% CI: 0.640–0.889, Brier score = 0.153). By decision curve analysis, the nomogram was shown to have a satisfactory net benefit between thresholds of 0.20 and 0.40. Ki-67, ultrasound volumetry, and scintigraphy techniques can play important roles in evaluating RAI therapy outcome in Graves‘ disease patients. The prediction nomogram shows reasonable accuracy in predicting NHRH.

## Introduction

The prevalence of hyperthyroidism is about 1.2% (1, 2). Graves’ disease (GD) accounts for 70–85% of the cases (3, 4). The manifestations of GD consist of signs and symptoms of systemic hypermetabolism, diffuse goiter, and orbitopathy (5). Therapies for GD include antithyroid drugs (ATDs), thyroidectomy, or radioactive iodine (RAI) therapy. ATD has only a 50% success rate for most patients after 1 year of treatment, and it may cause side effects such as agranulocytosis, aplastic anemia, acute and subacute liver necrosis, and cholestasis (6). On the other hand, the incidence of adverse reactions with surgical treatment is as high as 24% (7). Due to its rare side effects and high efficiency, RAI therapy has been recognized as the first-line treatment for GD in many parts of the world, including the United States (6). The principle of RAI administration is to optimize the therapeutic outcome while minimizing the residual radiation dose (8). There are two approaches for determining the administered RAI therapy activity dose: the calculation formula based on gland size and iodine uptake or the use of a fixed activity dose (9). However, 8% of patients still suffer from nonhealing or recurrence of hyperthyroidism (NHRH) after RAI therapy (10). The reasons for treatment failure are insufficient dose or poor response to radiation therapy (11). Evaluation of treatment response before administration is conducive to more accurate calculation of dose and reduction of NHRH. Multiple factors, such as age, gender, prior use of ATD, and the clinical course of the disease, have been believed to be associated with the therapeutic outcome of RAI therapy in GD patients by affecting treatment response (12, 13). However, it is unclear how to assess treatment response to radiation and predict the probability of NHRH.

RAI therapy for GD operates on a mechanism akin to external beam radiation therapy for tumors. The emission of beta-rays by ^131^I inflicts damage upon thyroid cells, triggering subsequent cell death (3). Consequently, there is a reduction in thyroid hormone synthesis and secretion. Some researchers posit that the response of GD patients to RAI therapy correlates with the sensitivity of thyroid follicular cells to beta-rays (14).

Ki-67, a cellular proliferation marker, has been employed to gauge the sensitivity of tumor cells to radiotherapy (15). The expression of Ki-67 indicates cells in the proliferation phase, predicting a favorable response to chemotherapy and radiotherapy (16, 17). Domoslawski *et al.* (18) demonstrated significantly higher Ki-67 expression in thyroid follicular cells of GD patients compared to patients with nodular goiter. Whether this heightened Ki-67 expression in follicular epithelial cells of GD patients forecasts a positive response to RAI therapy and, consequently, a reduced likelihood of NHRH remains unexplored.

The volume and hardness of the thyroid gland are pivotal reference indices for dose calculation (9). Three-dimensional ultrasound imaging technology offers a more precise measurement of thyroid volume compared to conventional ultrasound, with high consistency and repeatability (19). In contrast to clinical palpation, ultrasound elastography is recognized for quantitatively measuring and reflecting differences in thyroid hardness in GD patients (20). This paper introduces what appears to be the inaugural prediction model incorporating Ki-67 and ultrasound indices.

The objectives of this study were two-fold: first, to investigate the predictive value of Ki-67 and ultrasound in determining the outcomes of RAI therapy for GD patients and, second, to construct a prediction model. This endeavor seeks to establish a more evidence-based approach for developing individualized therapies, thereby enhancing the prospects of remission for patients.

## Materials and methods

This two-way cohort study was approved by the Research Ethics Committee of Zhangzhou Affiliated Hospital of Fujian Medical University (protocol no. 2020KYB138). All patients provided written informed consent.

### Patients

A total of 162 GD patients admitted to the Nuclear Medicine Department of our hospital from January 2020 to May 2022 were included in the study, with the study concluding on June 1, 2023.

### Definition of outcome events after treatment

Outcome events were categorized into three types based on follow-up results:

Event 1 (NHRH): The patient's hyperthyroidism symptoms did not resolve or worsened, and the patient may require another round of RAI therapy or initiate treatment with antithyroid medications.

Event 2 (premature hypothyroidism): Serum-free thyroxine (FT4) was below normal, and thyroid-stimulating hormone was higher than normal within 1 year of RAI treatment.

Censoring event (euthyroidism or loss of follow-up): Signs and symptoms of hyperthyroidism completely disappeared, and serum FT4 returned to normal within 1 year, or the patient was lost to follow-up. The maximum follow-up time for each patient was 1 year, starting 1 month after treatment. Follow-up terminated under the following conditions: (i) occurrence of any event in patients, (ii) follow-up exceeding 1 year, or (iii) reaching the study deadline.

The therapeutic effect of patients was determined by physicians.

### Inclusion and exclusion criteria

Inclusion criteria were: (i) age over 18 years, (ii) clinical physician diagnosis of GD (7), (iii) no contraindication for RAI therapy, and (iv) availability of pathological and clinical follow-up data. Exclusion criteria were: (i) history of thyroid surgery, (ii) prior RAI therapy, (iii) presence of thyroid malignancy, (iv) incomplete pathological data with no Ki-67 results, and (v) less than a year of follow-up after RAI therapy without any events when the study reached the deadline.

### Variables

#### Ultrasound-guided fine-needle aspiration biopsy: Ki-67 results

Fine-needle aspiration biopsy of thyroid tissue guided by ultrasound, conducted 2 days before RAI, involved the assessment of the cell proliferation index using Ki-67 staining via a Roche automatic immunohistochemistry assay. Observation was conducted under a microscope (eyepiece diameter 22 mm) with high magnification (×400). Specimens with ≥200 cells per field of vision were qualified for analysis. For sections with more cells or evident Ki-67 hot spots, two to three fields containing areas with the highest positive rate were selected for counting. For sections with few cells or no significant Ki-67 hot spots, the visual field containing all follicular cells in the sections was chosen. The Ki-67 proliferation index was calculated as follows:


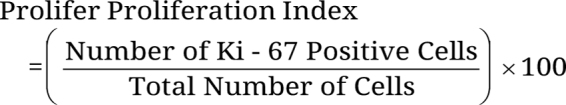



The results were then categorized into negative Ki-67 expression (proliferation index < 1%) and positive Ki-67 expression (proliferation index ≥ 1%).

#### Three-dimensional volume thyroid ultrasonography

The examination utilized an RAB-6 probe with a frequency of 7–14 MHz. Values were obtained through virtual organ computer-aided analysis at a rotation angle of 30° (six planes), and the thyroid contour was defined on each plane using the manual trace function. Three-dimensional images were automatically acquired by computer software, providing a comprehensive volume of the thyroid.

#### Dose of per-unit thyroid volume (DPUTV)

The activity of the RAI dose administered to the patient was determined based on gland volume and ^131^I uptake using a standard formula:

Empirical activity = 70−150 μCi per gram of thyroid tissue

DPUTV was calculated as follows:


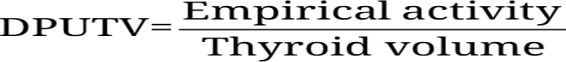



#### Shear wave elastic velocity value of the thyroid

**Figure 1:**
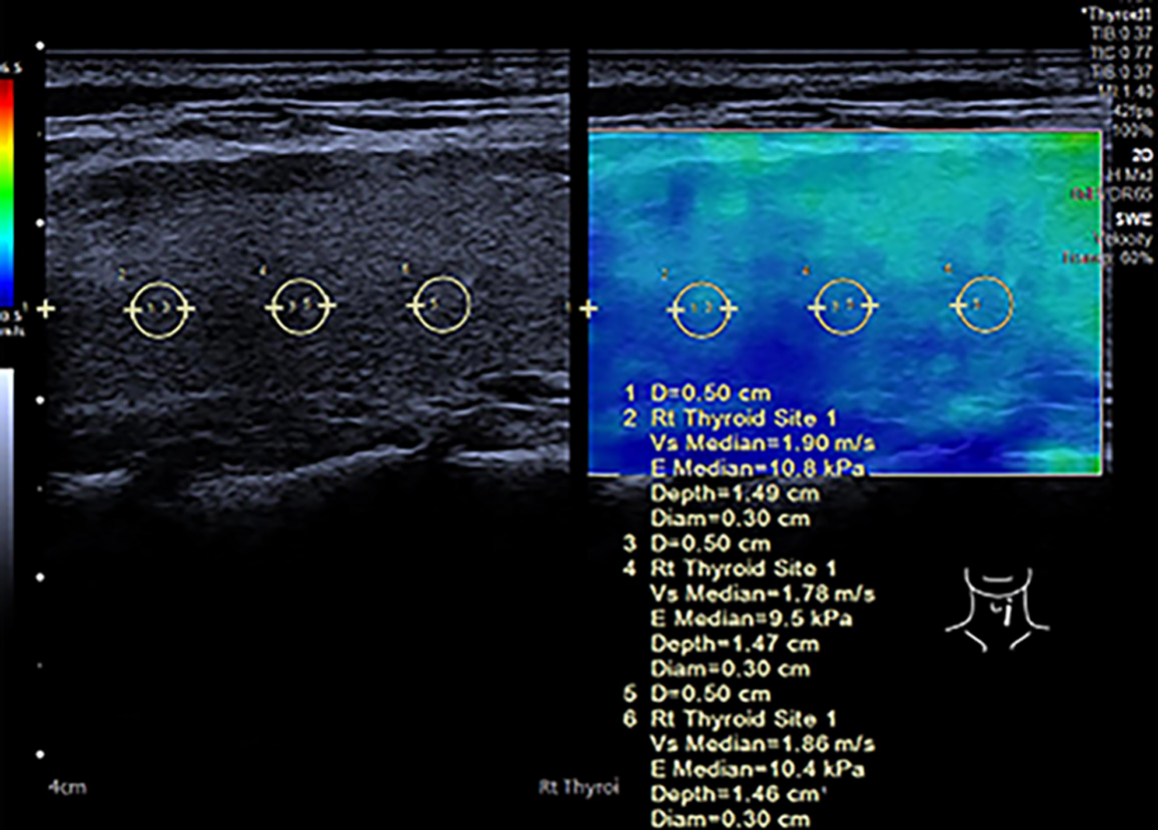
The diagram of shear wave elastic velocity value (SWE) of thyroid measurement. Under the elastic imaging mode, the maximum longitudinal section of one side of the thyroid was selected. SWE was measured once at 5-mm intervals at the center of the gland.

Thyroid shear wave elastography was conducted using the SEMENS ACUSON Sequoia 512 with a 10L4 linear-array probe. Under the elastic imaging mode, the maximum longitudinal section of one side of the thyroid was selected. Shear wave elastic velocity value (SWE) was measured at 5-mm intervals at the center of the gland, and the average value was calculated (Fig. 1).

#### Free thyroxine, free triiodothyronine, and thyrotropin receptor antibody

Levels of free triiodothyronine (FT3), free thyroxine (FT4), and thyrotropin receptor antibody (TRAb) in patients' venous blood were measured using an electroluminescent immunoassay (Beckman, DXI800) after overnight fasting. The reference ranges were: 3.10–6.80 IU/mL (FT3), 7.5–21.1 pmol/L (FT4), and < 0.15 mIU/L (TRAb), respectively.

#### Age, sex, and prior ATD use

Information on age, sex, and whether patients had taken ATDs before RAI therapy was obtained from clinical records.

### Statistical analysis

Statistical analyses were conducted using R Commander, version 4.2.1 (http://www.r-project.org/). Mean value interpolation was used to address missing values. Continuous data are presented as mean ± s.d. for normal distribution or as median (Q25, Q75) for non-normally distributed data. Categorical variables are presented as counts (*n*, %).

The competing risk model was employed to estimate multivariable-adjusted hazard ratios (HRs) and 95% confidence intervals (95% CIs) for NHRH. Variables with *P*-values less than 0.10 in univariate analysis were included in multivariate models for further analysis. Model selection was conducted based on the Akaike information criterion (AIC). The model‘s discriminating performance was assessed through receiver operator characteristic analysis, and its calibrating performance was evaluated using the Brier score. The model underwent internal validation through bootstrap resampling, and a nomogram was subsequently developed.

## Results

### Patients’ demographics and outcomes

Out of the initially selected 162 GD patients, 11 were excluded due to prior RAI treatment, 17 had incomplete pathological data with no Ki-67 results, and 7 had not experienced related events before the study deadline. Consequently, a total of 127 patients (91 women, mean age 44.2 ± 13.9 years) were included. Among these, 41 patients experienced NHRH, 73 had premature hypothyroidism, 4 achieved euthyroidism, and 9 were lost to follow-up.

### Cumulative marginal risk probability of NHRH after ^131^I treatment

**Figure 2:**
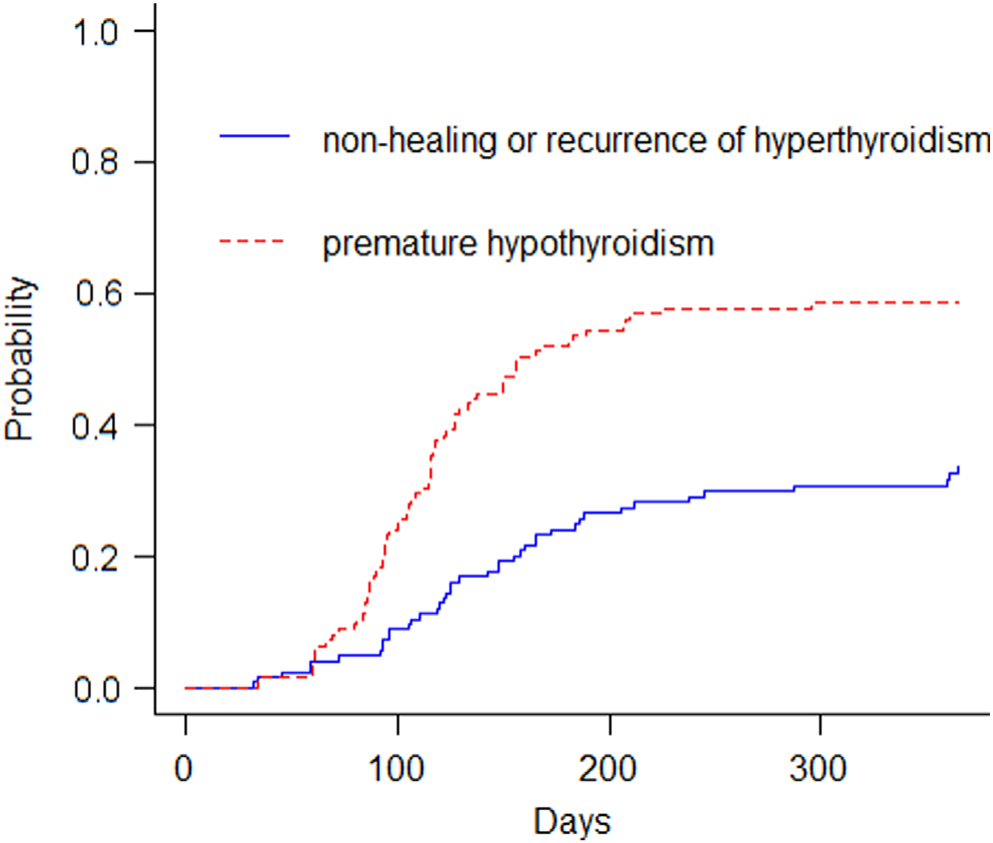
Cumulative marginal risk probability of nonhealing or recurrence of hyperthyroidism or premature hypothyroidism after radioactive iodine therapy.

Cumulative risk marginal probability analysis revealed that the probability of NHRH was approximately 25% after 300 days of ^131^I treatment (Fig. 2).

### Factors associated with NHRH

**Figure 3:**
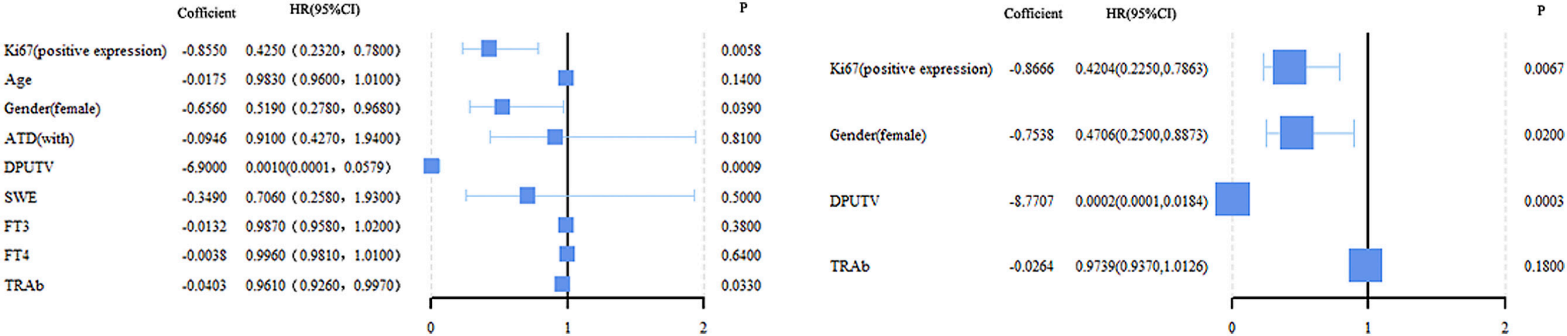
Association between clinical and pathological factors and NHRH in the competing risk analysis. ATD, the history of patients taking ATD before RAI therapy; DPUTV, dose of per-unit thyroid volume; SWE, shear wave elastic velocity value.

Positive Ki-67 expression, higher DPUTV, and female gender were associated with lower risks of NHRH (all *P* < 0.05). The HRs (95% CIs) were 0.42 (0.23, 0.79), 0.01 (0.00, 0.02), and 0.47 (0.25, 0.89), respectively (Fig. 3).

### Establishment and validation of the NHRH model

Based on the AIC, the competing risk model with the lowest AIC value (364.85) was obtained, calculating the score as follows:

Points = −0.87 × *X*1 − 8.77 × *X*2 − 0.75 × *X*3

where *X*1 is Ki-67 (positive = 1, negative = 0), *X*2 is DPUTV, and *X*3 is gender (female = 1, male = 0).

Internal validation of the model, using 1000 bootstrap resampling techniques, showed that the 95% CIs of the Ki-67, DPUTV, and gender coefficients were −1.728, −0.394, −13.970, −4.122, and −1.430, −0.114, respectively. The AUC of the model was 0.615 (95% CI, 0.250–1.000), 0.772 (95% CI, 0.640–0.889), 0.741 (95% CI, 0.608–0.852), and 0.717 (95% CI, 0.572–0.836) on 90, 180, 270, and 360 days after treatment, respectively. The validation also showed that the Brier score on 90, 180, 270, and 360 days after treatment was 0.046 (95% CI, 0.007–0.093), 0.153 (95% CI, 0.105–0.209), 0.184 (95% CI, 0.136–0.255), and 0.198 (95% CI, 0.147–0.272), respectively.

### Decision curve analysis

**Figure 4:**
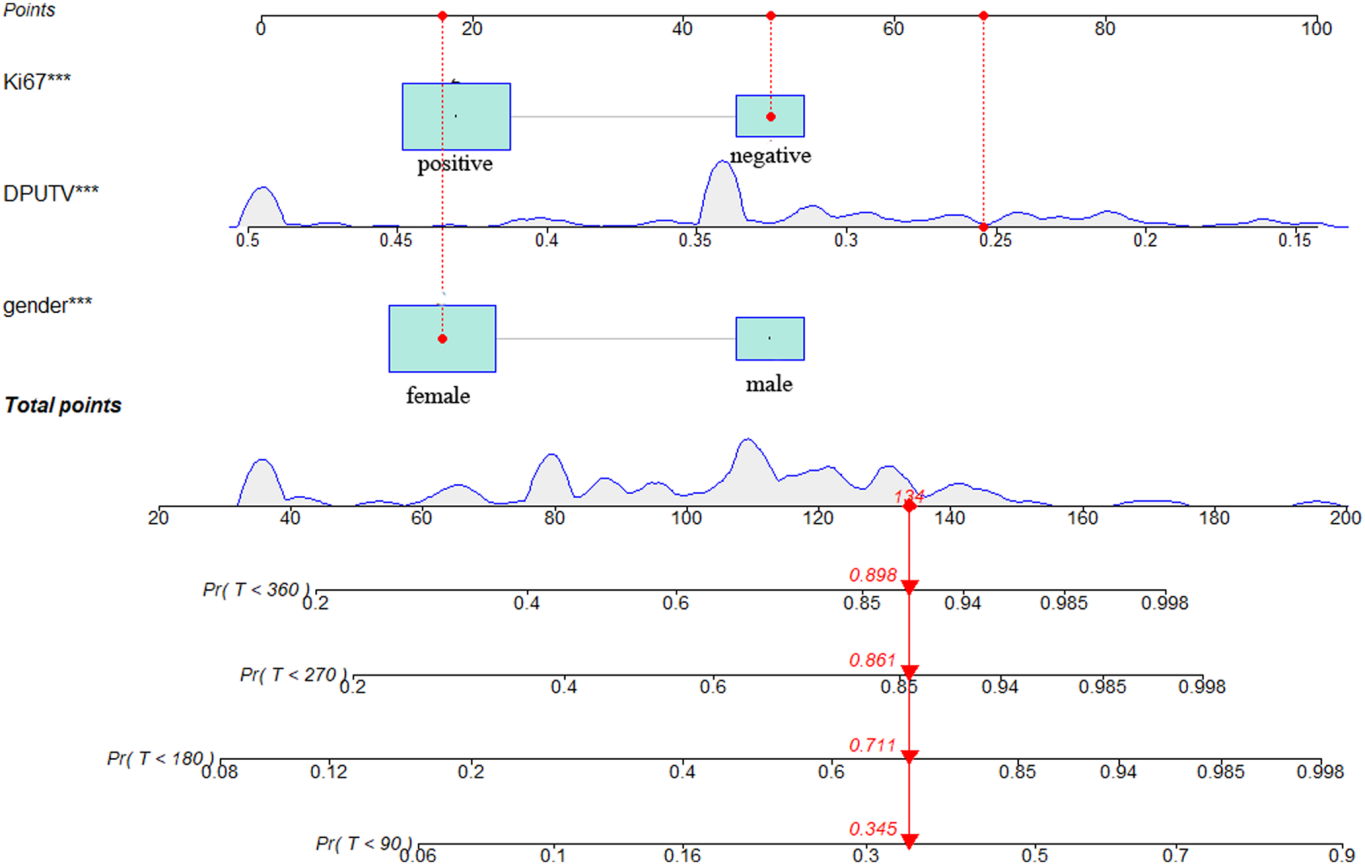
Prognostic nomogram used to predict the probability of cumulative risk for NHRH in GD patients when they were at 90, 180, 270, and 360 days after radioactive iodine therapy. In this study, patient no. 9 was confirmed to have nonhealing hyperthyroidism on the 132nd day after radioactive iodine therapy. The normogram score of this patient was 124, and his cumulative risks of NHRH predicted by nomogram were 34.5, 71.1, 86.1, and 89.8% at 90, 180, 270, and 360 days, separately, respectively. DPUTV, dose of per-unit thyroid volume.

**Figure 5:**
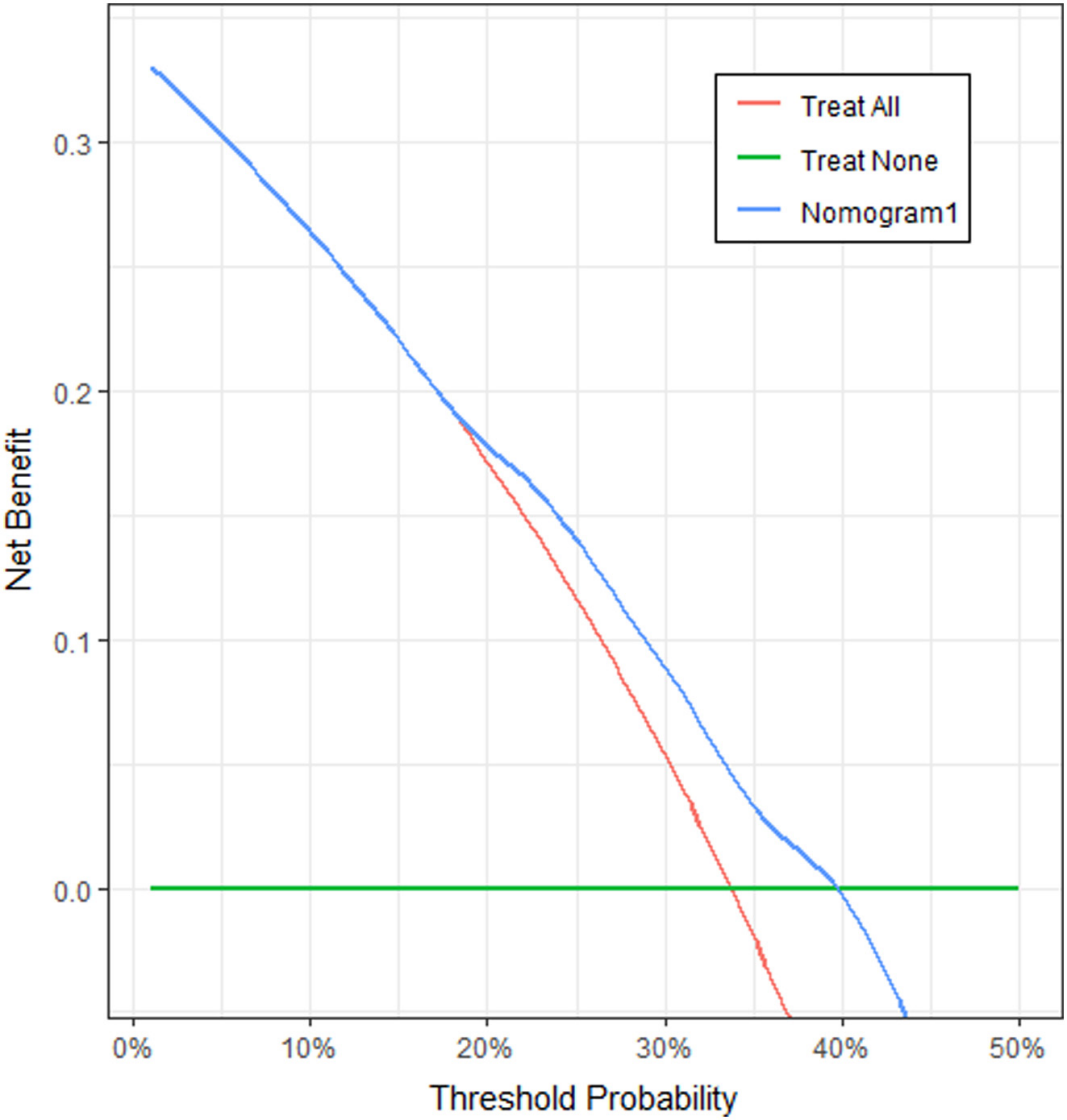
Decision curves for the competing risk model. The *y*-axis measures net benefit, which is calculated by summing the benefits (true positives) and subtracting the harms (false positives). Horizontal green line = net benefit when all patients are considered as not having NHRH and obliquered line = net benefit when all patients are considered as having NHRH. Compared with simple strategies (horizontal green line and oblique red line), the nomogram has a higher net benefit.

The nomogram, based on the model, was established to predict the probability of NHRH (Fig. 4). Decision curves for the nomogram's ability to predict the probability of NHRH after RAI therapy are presented in Fig. 5. It demonstrated applicability when thresholds ranged between 0.20 and 0.40.

## Discussion

In our study, cumulative risk marginal probability analysis indicated a 25% probability of NHRH after 300 days of RAI therapy (Fig. 2). NHRH in GD patients post RAI therapy signifies treatment failure and can significantly impact patients with hypermetabolic syndrome. To enhance treatment success rates, evaluating factors associated with NHRH and establishing a predictive model are crucial.

Previous studies explored factors such as age, sex, GD disease duration, history of ATD before RAI therapy, RAI dose, half-life, and TRAb positivity before treatment (10, 11). However, controversies persist among these studies. Our study identified Ki-67, DPUTV, and gender as factors associated with NHRH (Fig. 3). The results suggested that, under similar conditions, men are more prone to developing NHRH compared to female patients, consistent with Allahabadia *et al.* (21). DPUTV emerged as a protective factor against NHRH, indicating that a higher dose of ^131^I relative to the thyroid weight correlates with a lower risk of NHRH, aligning with previous findings (22, 23).

Our results indicate that GD patients with Ki-67-positive expression in thyroid follicular epithelial cells have a lower risk of NHRH. This could be attributed to the proliferative state of thyroid follicular cells expressing Ki-67, making them more sensitive to ^131^I radiation and leading to increased cell damage (15, 16). Therefore, Ki-67 may serve as an indicator reflecting the sensitivity of thyroid follicular cells to RAI therapy. Some studies highlight the significance of pre-RAI levels of FT3, FT4, and TRAb in predicting NHRH, showing inconsistency with our findings and warranting further investigation (24, 25). Interestingly, SWE demonstrated no correlation with treatment outcomes in our study, a finding that merits further verification.

Utilizing the AIC, we developed a competing risk model incorporating Ki-67, DPUTV, and gender. The model underwent validation with 1000 bootstrap resampling techniques, demonstrating reliable coefficients within their respective CIs. AUC and Brier score assessments confirmed adequate discrimination and calibration. Notably, the model exhibited its highest predictive accuracy at 180 days post therapy (AUC: 0.772). This suggests its potential utility in clinical practice for predicting cumulative NHRH risk at the 180-day mark. The decision analysis curve of the nomogram, based on the model, indicated a satisfactory net benefit for thresholds between 0.20 and 0.40 (Fig. 5), suggesting that if a patient‘s NHRH risk probability exceeds 20%, clinicians should consider more potent treatments.

Based on our results, conducting a fine-needle aspiration biopsy to assess Ki-67 expression in thyroid follicular cells before treatment may aid in selecting appropriate therapy for GD patients. However, given the invasive nature of this procedure, further research is needed to determine whether it should be universally performed before surgery. Additionally, we recommend employing three-dimensional volume thyroid ultrasonography to assess thyroid volume. Utilizing the nomogram proposed in our study, physicians can estimate patient outcomes before treatment and develop individualized therapies to enhance the likelihood of remission.

The main limitations of this study include a small sample size and the absence of external validation. We are committed to expanding the sample size and conducting multicenter studies to validate the related factors and the predictive model we have developed.

## Conclusion

Ki-67 and ultrasound techniques, including three-dimensional volume measurement and ultrasound-guided puncture, emerge as crucial factors in evaluating RAI therapy outcomes in GD patients. The prediction nomogram, based on a competing risk model, demonstrates reasonable accuracy in predicting NHRH.

## Declaration of interest

The authors have no relevant financial or non-financial interests to disclose.

## Funding

This work was supported by the funding of Naturehttp://dx.doi.org/10.13039/501100020487 Science Foundation of China (grant no. 82071943).

## Ethics approval

This two-way cohort study was approved by the Research Ethics Committee of Zhangzhou Affiliated Hospital of Fujian Medical University (protocol no. 2020KYB138).

## Consent to participate

Informed consent was obtained from all individual participants included in the study.

## Author contribution statement

Allauthors contributed to the study conception and design. All authors read and approved the final manuscript.
